# Influence of Imposed Strain on Weldability of Dievar Alloy

**DOI:** 10.3390/ma17102317

**Published:** 2024-05-14

**Authors:** Josef Izák, Marek Benč, Lenka Kunčická, Petr Opěla, Radim Kocich

**Affiliations:** 1 Faculty of Mechanical Engineering, Brno University of Technology, Technická 2896, 616 00 Brno, Czech Republic; 2 Department of Metallurgical Technologies, Faculty of Materials Science and Technology, VŠB Technical University of Ostrava, 17. Listopadu 2172-15, 708 00 Ostrava, Czech Republic; marek.benc@vsb.cz (M.B.); petr.opela@vsb.cz (P.O.); 3 Institute of Physics of Materials, Czech Academy of Science, Žižkova 22, 616 00 Brno, Czech Republic

**Keywords:** rotary swaging, heat treatment, weldability, tool steel, Dievar, microstructure

## Abstract

The presented work is focused on the influence of imposed strain on the weldability of Dievar alloy. Two mechanisms affecting the microstructure and thus imparting changes in the mechanical properties were applied—heat treatment (hardening and tempering), and rotary swaging. The processed workpieces were further subjected to welding with various welding currents. In order to characterize the effects of welding on the microstructure, especially in the heat-affected zone, and determine material stability under elevated temperatures, samples for uniaxial hot compression testing at temperatures from 600 to 900 °C, optical and scanning electron microscopy, and microhardness testing were taken. The testing revealed that, although the rotary swaged and heat-treated samples featured comparable microhardness, the strength of the swaged material was approximately twice as high as that of the heat-treated one—specifically 1350 MPa. Furthermore, it was found that the rotary swaged sample exhibited favorable welding behavior when compared to the heat-treated one, when the higher welding current was applied.

## 1. Introduction

The continuous development of new materials in various industries is primarily driven by the ever-increasing demand. This trend is evident not only from the design of specialized processing methods, such as thermomechanical processing (TMP) of well-known metallic materials [1,2,3,4], but also from the development of new preparation processes, such as additive manufacturing [5,6,7]. Combinations of additive manufacturing and TMP have also been proposed, and their effects studied [8,9,10]. Also among research trends is the effort to develop a way of processing tool steels, which would simultaneously impart favorable strength properties and preserve advantageous weldability. Although it is also possible to achieve an increase in the mechanical properties of these steels via alloying, this way is not preferred due to issues related to preserving required weldability.

One of the highly favorable and widely used possibilities to achieve the mentioned goal is to perform a heat treatment. Among the implemented methods, for example, is increasing the austenitizing temperature [11,12], which, on the other hand, typically results in an undesired coarsening of the austenitic grains, which reduces the notch toughness and material plasticity (i.e., the characteristics important for subsequent processes steps, such as welding/hard-facing). The main reason behind this phenomenon is typically the time at the quenching temperature, which does not enable the growth of new (refined) austenitic grains [12]. After subsequent tempering, the hardness decreases due to the transformation of tetragonal martensite to tempered martensite featuring decreased levels of internal stress [13]. During tempering, which can also be performed in multiple stages, precipitation of carbides of VC, Cr_7_C_3_, Cr_23_C_6_, Mo_2_C, and Mo_6_C types occurs simultaneously. This can subsequently contribute to dislocation strengthening, as they present obstacles for dislocation movement [14,15,16]. Increasing the number of tempering cycles typically means increasing the amount of carbides in the structure [13]. From the viewpoint of fatigue strength, the most favorable results are typically achieved after a second tempering [17]. Several researchers have also studied the resistance of tool steels against abrasive wear after performing combinations of cryogenic treatment and tempering. For example, Dhokey et al. [18] stated that, with a higher tempering temperature, the number of precipitated carbides increased, and thus also increased the resistance to abrasive wear.

Another way to improve the mechanical properties of metallic materials is to apply methods of plastic deformation, possibly in combination with thermomechanical processing (TMP) (e.g., [19,20,21]). Contrary to the above-mentioned way, the materials (i.e., steels) typically preserve their favorable plasticity, which is advantageous not only from the viewpoint of weldability [22,23]. It is a known fact that plastic deformation typically leads to increased strength due to grain size decrease (according to the Hall–Petch relation) [24,25]. The positive effects of plastic deformation on refining the microstructures and thus affecting the material properties have been studied by many (e.g., Hlaváč et al. [26], Kocich et al. [27], Straumal et al. [28], Valiev et al. [29], or Kunčická et al. [30]). Up to now, numerous methods of processing, that can be used to prepare UFG (Ultra-Fine-Grained) structures, nanostructures, or gradient structures, have been developed; these methods are severe plastic deformation (SPD) processes, or intensive plastic deformation (IPD) processes. The first mentioned group primarily features methods limited to the processing of samples of relatively small volumes (e.g., the equal channel angular pressing method and its modifications [31,32,33], high pressure torsion [34,35], friction stir processing [36], etc.) In the case of the second group of methods, the volumes of the processed samples are virtually unlimited. One of the IPD processes is rotary swaging (RS), which advantageously imposes shear strain increments to the workpiece and thus enables the processing of materials with limited formability [37,38,39,40,41]. As it is a (near) net shape forming technology, which can be used to commercially produce long products of various cross-sectional geometries with no need for further machining, it is a promising processing method even for tool steels.

Dievar alloy represents one of the most commonly used materials in the industry due to its excellent mechanical properties (high strength and hardness) and resistance to thermal stress [42,43,44]. The most common application of Dievar tool steel is for tools operating at elevated temperatures, i.e., for components used for forging, rolling, extrusion, etc. [45,46,47]. Such tools or components are typically subjected to wear and can exhibit tearing. Therefore, in order to improve their functional properties, they are usually treated by hard-facing. Up to now, numerous methods of hard-facing, that can also be used for tool renovation, have been developed; these methods are, e.g., arc welding technologies, or specialized hard-facing technologies. The first group comprises typical methods during which the heat for melting and depositing the weld metal is generated by electric arc (e.g., shielded metal arc welding [48,49,50], gas metal arc welding [51,52], gas tungsten arc welding [53,54], flux-cored arc welding [55], submerged arc welding [56,57], etc.), while the second group of methods uses a concentrated energy source (e.g., plasma [58,59], laser [60,61], or electron-beam welding [62], etc.) One of the above-mentioned technologies is gas tungsten arc welding, which is advantageously utilized due to its high weld quality and low dilution. Therefore, it is suitable for the deposition of heterogenous materials. Numerous works focused on preparing heterogenous weld overlays to enhance the functional properties or extend the lifespan of various components have been published. The studied hard-faced materials have been, for example, based on Fe [45,57,60], Co [45,51,53], Ni [53,54,60], or W [63].

The research presented herein focuses on the influence of the selected processing method and consequent material strengthening on the weldability of Dievar alloy. The primary aim of this study is to investigate the effect of selected welding parameters on structures prepared by heat treatment and/or rotary swaging. For comparison of the structures, mechanical properties, and weldability, samples were also prepared using a method conventionally used to enhance the strength and hardness of the alloy through heat treatment (hardening and tempering). The method of increasing strength and hardness by hardening and tempering is well known. The weldability of tool steels in hardened and tempered condition has been the subject of numerous publications (e.g., [50,53,54]). The majority of these focus on the renovation of tools for processing (forging, extrusion, etc.), using materials based on Co, Ni, and other alloys, as described above. The primary innovation of the method presented herein is that increasing the strength of the tool steel is achieved via the rotary swaging method. As far as the authors’ knowledge reaches, no such studies discussing the effects of rotary swaging on the structures and behaviors of tool steels with respect to their weldability have been published. Assessing the influence of the processing method and optimizing its effects on the structure and properties is a good incentive for research to move in this direction. The observations are primary performed in the heat-affected zone (HAZ), where there is an increased risk of exhibiting the formation of brittle structures and crack formation. Structure development for the selected welding parameters without preheating is also assessed. Deepening knowledge in this area is crucial to improve welding/hard-facing processes and achieve optimal results for industrial practice. Therefore, determining the weldability of materials with such properties, i.e., strength and microhardness, with respect to degradation of HAZ, crack formation and propagation, utility properties, etc., is a significant challenge.

## 2. Materials and Methods

The experimental material was tool steel known under the trade name Dievar (Böhler-Uddeholm, Vienna, Austria). Its chemical composition is similar to that of the AISI H13 tool steel. The original diameter of the bar was 41 mm. The chemical composition analysis of the bar was conducted using optical emission spectrometry (OES) Q4 Tasman (Bruker-Quantron, Bremen, Germany) and the results are summarized in Table 1. During the analyses, the valence electrons of the atoms within the samples were excited by a high-energy spark discharge in an argon atmosphere. The measurement results were processed using ELEMENTAL.SUITE software (ver. QBS-8). For the chemical composition analysis of each supplied bar, five test samples with diameter of 41 mm and length of 10 mm were taken and the individual measurements were then averaged.

**Figure 1:**
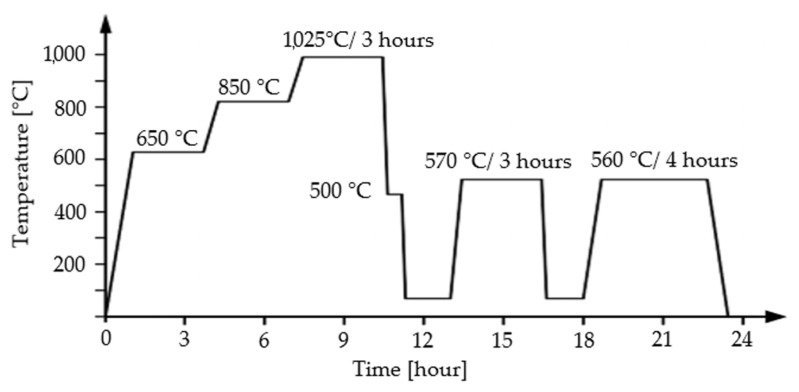
Hardening and tempering process.

The base material was soft annealed at a temperature of 850 °C for two hours and cooled to 600 °C with a cooling rate of 10 °C/s and then allowed to cool freely in air before austenitization. Then, the base material for welding was prepared in two different ways; the first one was hardening and tempering (HT), as depicted in Figure 1, and the second one was rotary swaging (RS).

For hardening, the material was machined from the original diameter of 41 mm to a diameter of 17.5 mm. Hardening consisted of three steps: heating, austenitization, and quenching. Heating was performed in two steps, at temperatures of 650 °C and 850 °C, in order to homogenize the temperature in the core and surface. Further, austenitization at the temperature of 1025 °C with the holding time of 3 h for transformation to fully austenitic structure. Then, samples were quenched. The cooling rate was 20 °C/s (between 800 °C and 500 °C, i.e., taking 15 s). Further, two tempering processes followed, 570 °C/3 h, and of 560 °C/4 h.

The second technology used for strengthening was muti-pass deformation via rotary swaging. The preheating temperature for each pass was 900 °C for 20 min. Swaging was carried out in individual passes reducing the original diameter of 41 mm to a final swaged diameter of 17.5 mm. The swaging ratios after each swaging pass were calculated via Equation (1):

(1) $$\varphi =\ln\left(\frac{S_{i}}{S_{n}}\right)$$

where *S_i_* and *S_n_* represent the cross-section areas at the input and output of the swaging dies, respectively. The total applied swaging ratio was 1.7.

**Figure 2:**
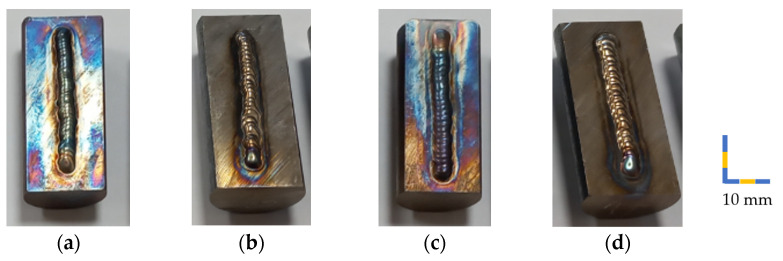
Welded samples: (**a**) No. 1 (50 A); (**b**) No. 2 (50 A); (**c**) No. 3 (70 A); (**d**) No. 4 (70 A).

For welding, the samples were cut to a length of 40 mm and halved in the middle of the base material (17.5 mm). Table 2 depicts the treatment of each sample prior to welding, i.e., quenching, tempering (HT), and rotary swaging (RS). Table 2 also shows the welding parameters (welding current, voltage, and speed). After processing, visual inspection was carried out at first, as documented in Figure 2a–d. The samples did not exhibit any signs of cracking, but discoloration of some samples (Figure 2a,c) could be observed. This could have occurred due to the lower thermal conductivity compared to the rotary swaged samples, as seen in Figure 2b,d.

The material for experiments was prepared using the tungsten inert gas (TIG) technology employing a TTP 220 AC/DC machine (WELCO spol. s.r.o., Uherský Brod, Czech Republic); the applied polarity was DC. All the samples were welded using a WL 20 tungsten electrode with a diameter of 1.6 mm and ground to an angle of 15–20°. Argon gas with a purity of 4.6 (99.996%) with a gas flow rate of 6 L/min during welding was utilized for the experiment. To monitor the HAZ, dilution area, and structural changes, a wire of the same chemical composition as the base material was employed for welding (to reduce the diffusivity of individual elements—primarily carbon). For reference, the chemical compositions of the base material and TIG wire are provided in Table 1. After welding, samples were cooled in silica sand.

Uniaxial hot compression testing procedure, which is usually conducted to evaluate hot deformation behavior of various materials via measuring flow stress responses, was further performed. The HT conventionally manufactured Dievar rod and the rotary swaged Dievar rod were processed into form of compression test samples with diameter of 11 mm and length of 16 mm. The prepared samples were subjected to a series of uniaxial compression tests using a Gleeble 3800 thermal-mechanical simulator (Dynamic Systems, New York, NY, USA). This testing was performed under four deformation temperatures (specifically 600, 700, 800, and 900 °C), i.e., 4 tests for conventional, HT, and RS materials sets. The test samples were heated directly to the specific deformation temperature with a heating rate of 10 °C·s^−1^ (realized via a direct electric resistance heating method) with a following dwell time of 5 min. After the preheating phase, compression with a strain rate of 0.1 s^−1^ was performed to the true (logarithmic) strain of −1.1. The temperature measurement was provided by a pair of K-type thermocouple wires welded in the middle length on the surface of each sample. Tantalum foils and nickel-based grease were utilized to protect the anvils and impede friction forces on the anvils–sample interface. The testing chamber was held under vacuum during the test to hinder oxidation processes.

Further, the samples were subjected to macroscopic observation using a ZEISS Neophot 32 microscope (Zeiss, Jena, Germany). The sizes of HAZ and dilution area were primary observed. Vilella’s reagent was used for visualization of the macrostructure.

The microstructure was observed by Keyence VHX-7000N digital optical microscope (OM, Keyence Corporation, Osaka, Japan) and TESCAN FERA3 GM (TESCAN, Brno, Czech Republic) scanning electron microscopy (SEM) equipment available at FZU, Institute of Physics of the CAS. The analysis of precipitates via scanning electron microscopy–energy dispersive spectrometry (SEM-EDS) was performed by Focused Ion Beam/Scanning Electron Microscope TESCAN LYRA 3 equipment at CEITEC Nano laboratories. Microstructure analysis was focused on observing the differences between HAZ and base material in the individual samples. The samples were mechanically ground on SiC papers and finally polished using OPS substance. Carpenter’s etchant was used for visualization of microstructure using optical microscopy.

The microhardness was measured with the load of 200 g (HV 0.2) using a Qness Q10A machine (Metalco Testing s.r.o., Roztoky u Prahy, Czech Republic). The microhardness was measured perpendicularly in section on all four samples through the weld metal (WM), HAZ, to the base material (BM).

## 3. Results and Discussion

### 3.1. Hot Compression Testing

**Figure 3:**
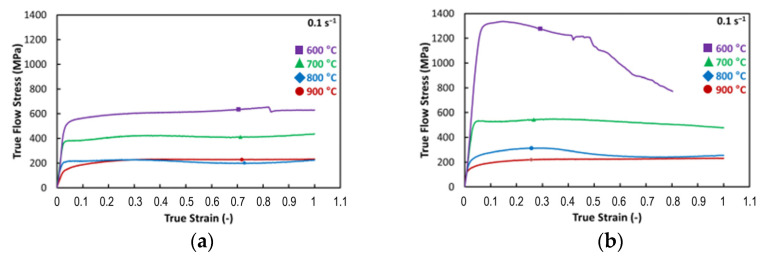
Flow stress evolution of: (**a**) HT and (**b**) RS Dievar alloy.

The above-described uniaxial compression test procedure enabled us to obtain experimental flow stress responses of both the examined material states, which are graphically expressed in Figure 3a,b for the conventional heat-treated and swaged samples, respectively. The testing was conducted with regard to material stability at elevated temperatures and to compare the stress properties between both the states. Additionally, the recrystallization temperature of the RS material can be seen in the graph in Figure 3b. The approximate recrystallization temperature was determined from the stress–strain curve (Figure 3b). The phenomenon of recrystallization can be characterized by a decrease in deformation as stress increases at this temperature. Therefore, the recrystallization temperature was approximately determined to be around 600 °C. However, for an exact determination of this value, more exact testing methods, such as differential scanning calorimetry (DSC), would have to be employed. This temperature is not only important for determining the temperature range in which material can be used, but also to design and characterize the annealing effects occurring during welding.

Han et al. [64] and Li et al. [65] reported that as-cast H13 steel at temperature of 900 °C and strain rate 0.1 s^−1^ exhibited material flow stress higher than 200 MPa. With respect to the higher testing temperatures (specifically 900 and 800 °C), the effects of RS and HT procedures are practically negligible in terms of the material strength. However, at temperatures of 700 °C and lower, the RS-modified Dievar structure starts to exhibit a significant increase in flow stress. It is noticeable that the lower the testing temperature, the more pronounced the increase in flow stress response. At the temperature of 700 °C, the flow stress of the rotary swaged material compared to the heat-treated material increased from 435 MPa to more than 500 MPa, i.e., exhibited a difference of 115 MPa. Considering the temperature of 600 °C, the flow stress response increased from about 650 MPa to more than 1300 MPa, which is an increase of 650 MPa.

### 3.2. Macrostructure Evaluation

**Figure 4:**
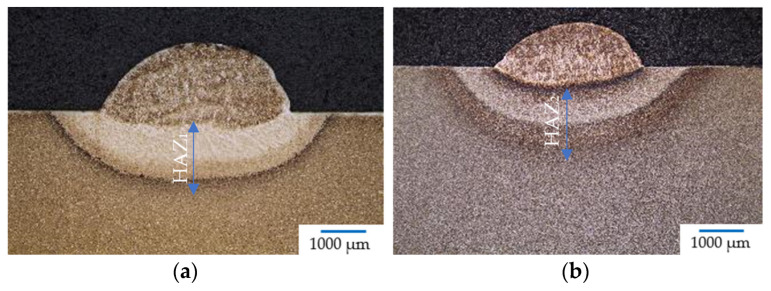

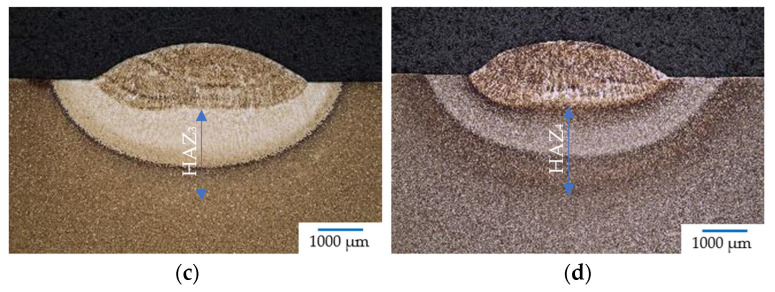
Macrostructure of sample: (**a**) No. 1 (50 A); (**b**) No. 2 (50 A); (**c**) No. 3 (70 A); (**d**) No. 4 (70 A).

The macrostructure of the examined samples can be seen in Figure 4a–d. No crack indications were observed in the macrostructure images of Figure 4a,d. HAZs were measured from the top of the base material, as depicted in the figures. Higher HAZ sizes were observed in the base materials subjected to rotary swaging (Figure 4b,d), compared to the samples subjected to heat treatment (Figure 4a,c). The HAZ sizes in the individual samples were: HAZ_1_ = 1.6 mm; HAZ_2_ = 2.0 mm; HAZ_3_ = 2.0 mm, and HAZ_4_ = 2.5 mm. Upon initial observation, it is evident that the heat-treated sample welded with 70 A (sample No. 3) has comparable HAZ size as the sample prepared using rotary swaging and welded with 50 A (sample No. 2). However, this does not necessarily mean that the rotary swaged samples are inferior. It depends on other parameters, which have significant influence on HAZ, such as structures and related microhardness.

During welding/hard-facing, an area known as the dilution zone arises due to the melting of the wire and base material. However, the aim is to minimize the dilution zone to prevent the formation of potentially dangerous phases during solidification, such as precipitation of brittle carbides, which can reduce the lifespan, create shrinkage voids, inclusion, etc. For samples welded at 70 A, prepared by both heat treatment and rotary swaging, the area of dilution was almost the same, i.e., sample No. 3 (47%) and sample No. 4 (49%). The dilution in sample No. 1 was 18% and in sample No. 2, it was 33%. The difference between these samples was rather significant (15%).

### 3.3. Microstructure Evaluation Using Optical and SEM Microscopy

**Figure 5:**
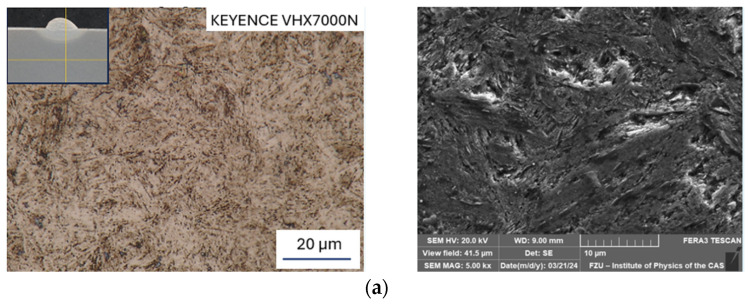

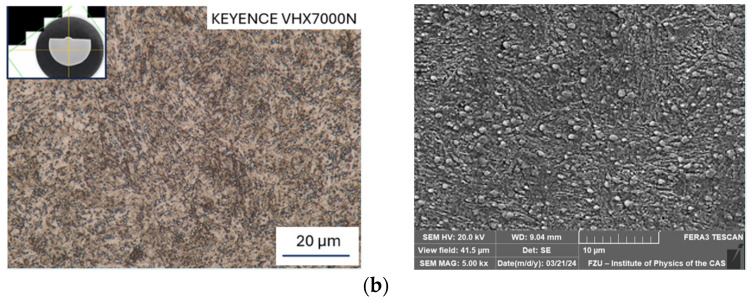
Microstructure of base material; OM, mag. 2000× (left) and SEM, mag. 5000× (right): (**a**) hardening and tempering; (**b**) rotary swaging.

The microstructures of the two strengthened base materials, i.e., after heat treatment and rotary swaging, can be seen in Figure 5a,b. The structure of the heat-treated material (Figure 5a) consists of fine sorbitic martensite with unevenly dispersed carbides. Carbides can be observed in Figure 5a (on the right). The carbides are either dispersed throughout the structure in the form of circular shapes with a size of 1 µm, or as clusters of finer carbides (see bright spots) with a size of 10 µm. Circular carbides are likely chromium-based, while cluster carbides are likely molybdenum based (as documented further).

The structure after rotary swaging (Figure 5b) exhibited a completely different morphology compared to that after heat treatment. It is composed of fine needle-like martensite (eventually bainite structure) with very finely and homogenously dispersed carbides measuring between 1 and 1.5 µm. A structure produced in this manner will not only feature advantageous strength, as demonstrated by uniaxial hot compression tests (Figure 3b), but will also exhibit superior resistance to abrasion and adhesive resistance [66]. Lange et al. stated that the primary reason for predominant failure of hot forging dies is the wear of the tool surfaces [67].

**Figure 6:**
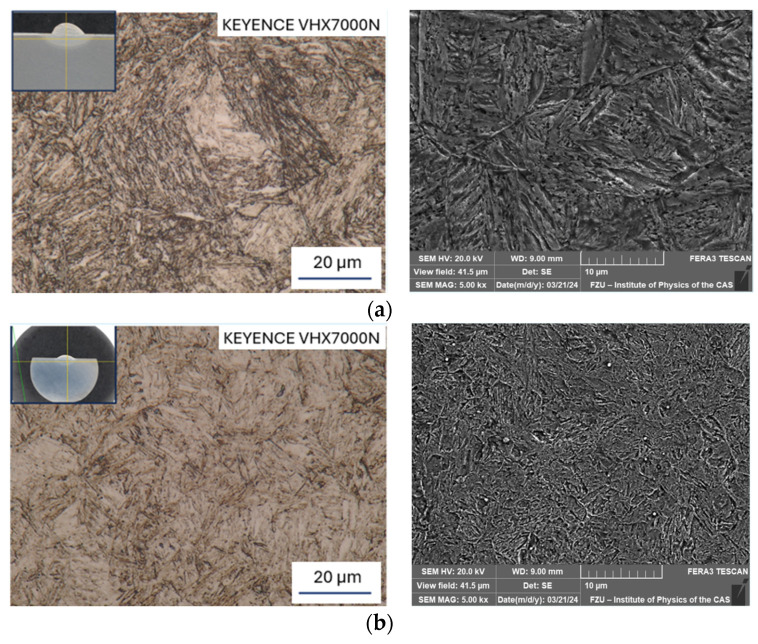

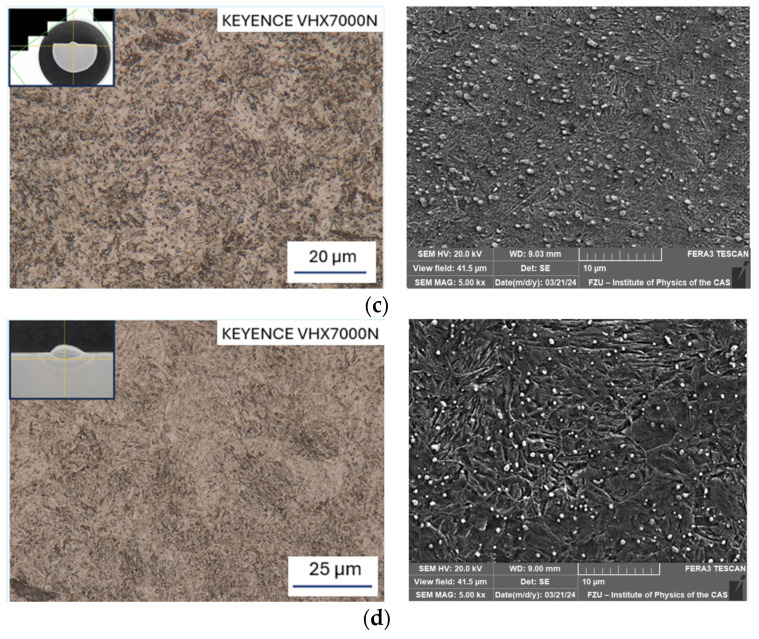
Microstructure of HAZ; OM, mag. 2000× (left) and SEM, mag. 5000× (right): (**a**) Sample No. 1 (50 A); (**b**) Sample No. 3 (70 A); (**c**) Sample No. 2 (50 A); (**d**) Sample No. 4 (70 A).

**Figure 7:**
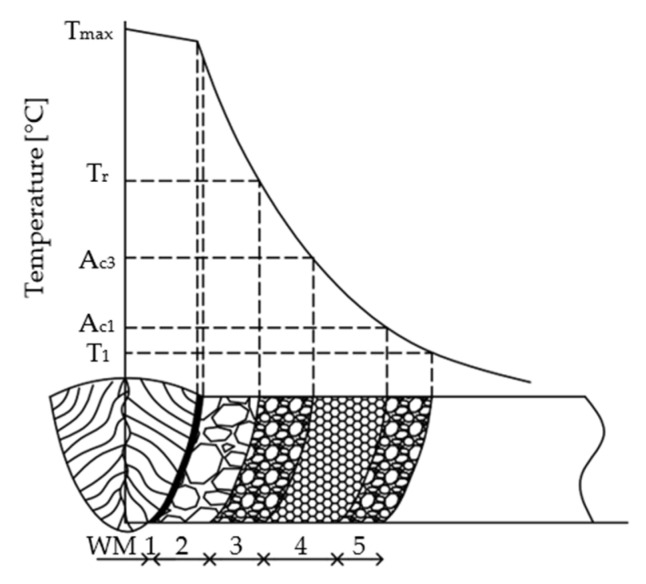
Diagram of the HAZ: (WM) weld metal; (1) fusion line; (2) overheated section FL–Tr; (3) grain-refined (normalized) section Tr–Ac_3_; (4) partly grain-refined section Ac_3_–A_C1;_ and (5) recrystallized section Ac_1_–T1.

Figure 6a–d depict the results of microstructure observations from the HAZs, specifically from the so-called highly overheated section (Figure 7). There is the highest risk during welding/hard-facing due to the formation of coarse-grained structures, resulting in increased microhardness and the possibility of initiation and propagation of (micro)cracks.

When comparing the base material in Figure 5a to the HAZ welded at 50 A (Figure 6a), the microstructure is composed of coarse plate martensite with a relatively lower number of carbide particles. The structure in the HAZ (Figure 6b), after welding at 70 A, is characterized by needle-like martensite with dispersed circular carbide particles, when compared to the base material (Figure 5a). The HAZ in Figure 6a is characterized by coarser martensite, when compared to the HAZ in Figure 6b.

When comparing the base material prepared via rotary swaging (Figure 5b) and the HAZ in Figure 6c,d, it can be observed that carbides are dissolved into the solid solution. Furthermore, it can be noticed that the structure of the HAZ welded at 70 A (Figure 6d) is coarser (needle-like martensite and original austenitic grain coarsening) than the structure welded at 50 A (Figure 6c). The carbides in Figure 6d are finer than in Figure 6c due to gradual dissolution into the solid solution.

The HAZ is composed of several zones, depicted in Figure 7, where the properties of the base material undergo changes depending on the temperature cycles during welding/hard-facing.

### 3.4. Analysis of Precipitates

**Figure 8:**
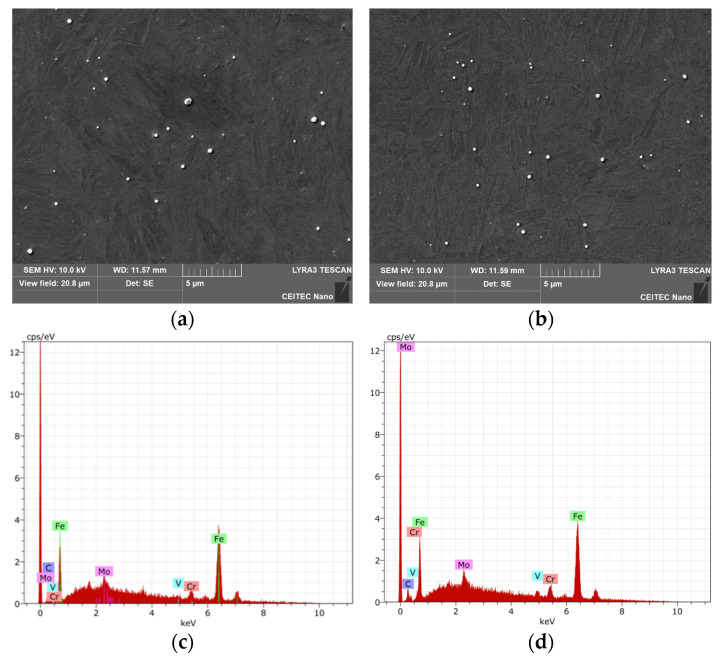
Sample No. 1: (**a**) Base material; (**b**) HAZ; (**c**) Chemical composition of base material; (**d**) Chemical composition of precipitate from base material.

Samples No. 1 (HT) and No. 4 (RS) were chosen for observation of precipitates. In the base material of sample No. 1 (Figure 8a), there were Mo-rich precipitates. In the HAZ (Figure 8b), there was partial dissolution of Mo back into the solid solution and, instead, there was a higher occurrence of Cr-rich precipitates. The chemical composition of the base material (EDX from the area of Figure 8a) was (in wt.%): Fe 90.5, Cr 4.8, Mo 2.2, V 1.6, and C 0.9 (determining C content using EDX is rather unreliable). The chemical composition spectrum of the area is shown in Figure 8c, while Figure 8d captures an example of the chemical composition of precipitate rich in Mo from the heat-treated base material. The acquired results confirmed the phenomena observed in the microstructure of sample No. 1 in Chapter 3.3.

**Figure 9:**
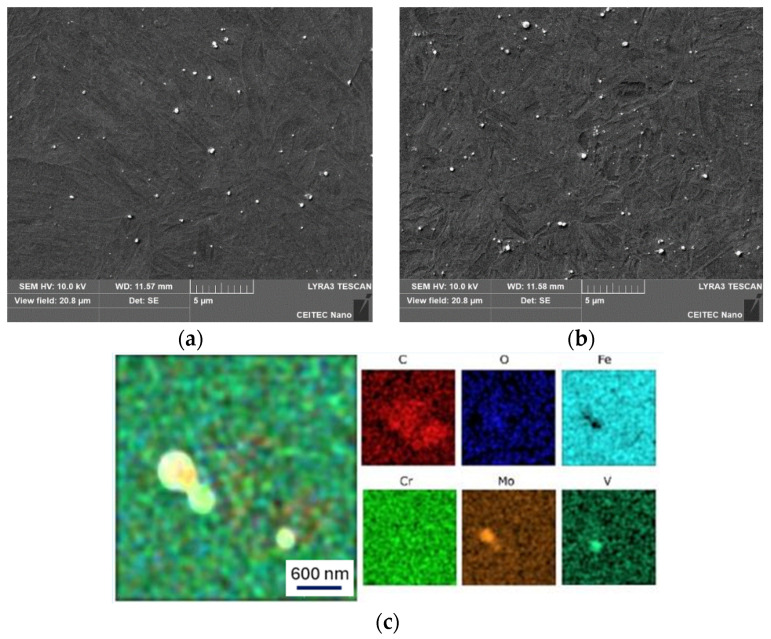
Sample No. 4: (**a**) Base material; (**b**) HAZ; (**c**) Map of chemical composition for base material.

In the base material of sample No. 4 (Figure 9a), precipitates were again present. However, they were notably finer compared to heat-treated samples, and contained high levels of alloying elements Mo, Cr, and V. Figure 9a shows the presence of two types of precipitates in the structure: finer and coarser ones. The coarser precipitates were rich in Mo and exhibited the following average chemical composition (in wt.%): Mo 47.4, Fe 33.7, Cr 5.5, V 2.6, and C 10.4. On the other hand, the finer precipitates were rich in V and had the following average chemical composition (in wt.%): V 37.8, Fe 21.7, Cr 5.1, Mo 10.5, and C 12.6. The average composition for each type of precipitate was measured from five random precipitates from a given micrograph (Figure 9a). The distribution of elements in the structure is also evident from the chemical composition map depicted in Figure 9c. After welding, i.e., in the HAZ, local clusters of fine Cr-rich precipitates formed in the structure, although coarser precipitates with higher alloy content (Mo and V) were still present, as shown in Figure 9b.

In the case of heat-treated steel, welding did not have a significant influence on the precipitates. However, the effect on the HAZ was noticeable for the material prepared by intensive plastic deformation. The processing promoted a more or less uniform distribution of the alloying elements in fine precipitates (V, Mo, Cr)C. After welding, there was partial dissolution of the alloying elements into the solid solution, and preferential precipitation of carbides rich in Cr (most probably the Cr_23_C_6_ type; however, deeper investigation via TEM would be necessary to confirm this presupposition) [68,69,70].

### 3.5. Microhardness Evaluation

**Figure 10:**
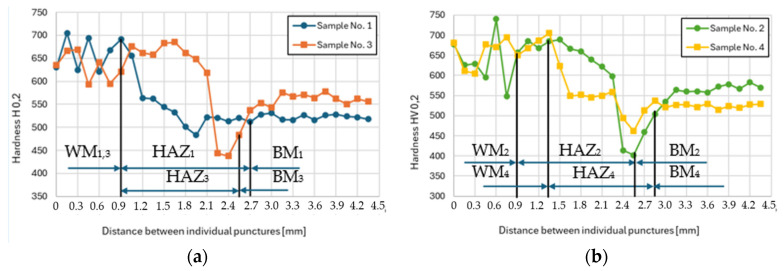
Measurement of microhardness: (**a**) Sample No. 1 and No. 3 (HT); (**b**) Sample No. 2 and No. 4 (RS); WM_x_ (weld metal), HAZ_x_ (heat-affected zone), BM_x_ (base material).

The results of microhardness measurements performed for each examined sample are summarized in Figure 10a,b. There are three distinguishable areas: weld metal (WM), HAZ, and base material (BM). The microhardness of the base material in both cases, i.e., HT and RS, was comparable: 540 ± 10 HV0.2, but the strength value at the elevated temperature was notably different; the material prepared via rotary swaging at 600 °C featured a tensile strength twice as high as the heat-treated one, i.e., 1350 MPa (Figure 3b). The weld metal had the same chemical composition for all the samples. Thus, under the same cooling conditions, it also had the same microhardness, which varied depending on the location of the indent in structure. The microhardness ranged between 550 and 740 HV0.2 (with an average microhardness of 650 HV0.2).

As previously mentioned, the crucial parameter for weldability is the structure and resulting mechanical properties in the HAZ (primarily microhardness). The critical zone is the so-called overheated section.

In sample No. 1 (Figure 10a), the overheated zone appears limited due to a low heat input into the base material and partial dissolution of Mo carbides (Figure 8b). Conversely, sample No. 3 (Figure 10a) exhibits a substantially enlarged overheated region extending from 0.9 to 1.65 mm. The higher temperature probably led to reaching the tempering temperature and because the sample was cooled in silica sand, there was enough time for carbides’ precipitation, thereby increasing microhardness. In the case of the rotary swaged sample welded at 50 A, the microhardness curve in the HAZ (Figure 10b) closely resembles that of welding at 70 A (Figure 10a). However, there is an opposite effect occurring here, where carbides dissolve into a solid solution during welding (Figure 6c). In sample No. 4 (Figure 10b), is visible that the overheated area diminishes due to the dissolution of carbides into a solid solution (Figure 6d and Figure 9b). This means that the microhardness in the overheated zone is also lower, which is favorable from a view of welding perspective. Furthermore, the HAZ follows the principles depicted in Figure 7, where a zone of normalization accompanied by a decrease in microhardness and recrystallization occurs. During the recrystallization, the microhardness in the HAZ is lower than the microhardness of the base material in all cases (sample No. 1 to No. 4).

## 4. Conclusions

The study investigated the influence of imposed strain on the weldability of Dievar alloy. Based on the acquired results, the following conclusions can be drawn:

- Heat-treated sample welded at 70 A featured comparable size of HAZ as sample processed by rotary swaging and welded at 50 A, i.e., 2 mm,
- Heat-treated sample exhibited coarser (martensitic) structure compared to rotary swaged one,
- Compared to heat-treated sample, the rotary swaged one showed uniformly distributed fine (Mo, Cr, V)C precipitates,
- Rotary swaged and heat-treated samples had comparable microhardness (540 ± 10 HV0.2),
- Rotary swaged sample had twice as high strength as heat-treated sample (RS 1350 MPa and HT 650 MPa),
- Rotary swaged material will have a higher thermal stability (at 600 °C) than the heat-treated material, due to the higher number of carbides preventing the movement of dislocations observed in base material structure,
- In the case of heat-treated sample, higher heat input led to precipitation of carbides and increase in microhardness in HAZ (specifically in the highly overheated zone),
- During welding of rotary swaged sample, higher heat input resulted in dissolution of carbides into the solid solution and decrease in microhardness in HAZ,
- Weldability of rotary swaged sample was superior to that of heat-treated one (based on the microhardness profile in heat-affected zone).

Further studies could investigate not only the mechanical properties of such materials, but also a deeper understanding of welding at higher current or weldability, for example using cold metal transfer (CMT) technology.

## Figures and Tables

**Table 1:** Chemical composition of base material Dievar and TIG wire (wt.%).

	C	Si	Mn	Cr	Mo	V	P	S	Fe
BM	0.33	0.17	0.38	4.93	2.00	0.47	0.0017	0.0005	Rest.
TIG wire	0.35	0.20	0.50	5.00	2.30	0.60	-	-	Rest.

**Table 2:** Sample identification, strengthening mechanism, and welding parameters.

Sample	Strengthening Mechanism	Welding Current [A]	Welding Voltage [V]	Welding Speed [mm/s]
No. 1	HT	50	10	0.82
No. 2	RS			1.34
No. 3	HT	70	13	1.44
No. 4	RS			1.80

## Data Availability

The original data supporting the research are available upon request from the corresponding author.
